# Global phylogeography of pelagic *Polynucleobacter* bacteria: Restricted geographic distribution of subgroups, isolation by distance and influence of climate†

**DOI:** 10.1111/1462-2920.12532

**Published:** 2014-07-15

**Authors:** Martin W Hahn, Ulrike Koll, Jitka Jezberová, Antonio Camacho

**Affiliations:** 1 Research Institute for Limnology, University of InnsbruckMondseestrasse 9, A-5310, Mondsee, Austria; 2 Biology Centre of the ASCR, v.v.i., Institute of HydrobiologyČeské Budějovice, Czech Republic; 3 Cavanilles Institute for Biodiversity and Evolutionary Biology, University of ValenciaBurjassot, Spain

## Abstract

The free-living planktonic freshwater bacterium *Polynucleobacter necessarius* subspecies *asymbioticus* (> 99% 16S rRNA similarity) represents a taxon with a cosmopolitan distribution and apparently ubiquitous occurrence in lentic freshwater habitats. We tested for intra-taxon biogeographic patterns by combining cultivation-independent and cultivation methods. A culture collection of 204 strains isolated from globally distributed freshwater habitats (Arctic to Antarctica) was investigated for phylogeographic patterns based on sequences of two markers, the 16S–23S internal transcribed spacers and the glutamine synthetase gene (glnA). Genetic distance between isolates showed significant geographic distance-decay patterns for both markers, suggesting that an isolation-by-distance mechanism influences the global phylogeography. Furthermore, a couple of subgroups showed restricted geographic distributions. Strains of one subgroup were exclusively obtained from tropical sites on four continents (pantropical subgroup). Cultivation-independent methods were used to confirm the restricted geographic distributions of two subgroups. The pantropical taxon could be detected in 63% of investigated tropical habitats but not in any of 121 European freshwater samples. Physiological tests indicated that almost all strains of the pantropical subgroup failed to grow at temperatures of 4°C, while strains affiliated with other subgroups showed good growth at this temperature. This suggests that thermal adaptation is involved in phylogeographic structuring of the global *Polynucleobacter* population.

## Introduction

An increasing number of studies suggests that the distribution of many microbial taxa shows biogeographic patterns (Bass and Boenigk, 2011; Fontaneto and Brodie, 2011; Hanson *et al*., 2012), but not much is known about the underlying mechanisms. Basically, such biogeographic patterns can result from barriers to dispersal of organisms (i.e. limited gene flow in combination with mutation, selection and/or drift, which causes divergence of populations) (Hanson *et al*., 2012). Alternatively, these patterns may simply reflect geographic distribution patterns of site-specific environmental conditions (Tamames *et al*., 2010), such as climate-determined features, habitats or host species suitable for maintaining populations of microbial taxa. It is not doubted that such environmental selection (filtering) of taxa by site-specific ecological conditions is significant in shaping microbial distribution patterns (Baas Becking, 2011; Crump *et al*., 2004; Gray *et al*., 2007). The role of dispersal barriers in biogeography of microorganisms is, however, controversial. A school of microbiologists argues that at least free-living (not host-associated) microorganisms differ from macroorganisms in the lack of any dispersal barriers or limitations (Baas Becking, 2011; Finlay, 2002; de Wit and Bouvier, 2006). This hypothesis is partially supported, for instance, by observations of long-distance or even intercontinental transport of microorganisms (Schlichting *et al*., 1978; Gorbushina *et al*., 2007; Hervàs *et al*., 2009; Pearce *et al*., 2009; DeLeon-Rodriguez *et al*., 2013). However, successful colonization of new habitats by microorganisms is a two-step procedure consisting of dispersal to and establishment of a population in the new habitat. The latter requires viable cells to be able to arrive (Gorbushina *et al*., 2007; Hervàs *et al*., 2009), to grow under the local environmental conditions (Hervàs *et al*., 2009) and to compete successfully with potential local inhabitants. Successful dispersal of microorganisms may thus be limited by pre- and post-colonization barriers (Marshall *et al*., 2010). Several investigations suggested that pre-colonization (dispersal) barriers exist for some microbial taxa (Papke *et al*., 2003; Whitaker *et al*., 2003), while other taxa seem not to be limited by dispersal (Gorbushina *et al*., 2007; van Gremberghe *et al*., 2011). On the other hand, knowledge on potential abiotic or biotic post-colonization barriers is very limited.

Biogeography studies on microorganisms tend either to compare entire communities or to focus on particular taxa (Hanson *et al*., 2012). Most community studies are based on a single locus and employ partial small subunit rRNA (16S or 18S rRNA) sequences as phylogenetic marker, resulting in a pronounced limitation in phylogenetic resolution due to the conserved nature of these ribosomal sequences. This limitation in phylogenetic resolution may result in a failure to reveal existing biogeography (Bass and Boenigk, 2011; Hanson *et al*., 2012). By contrast, investigations focusing on particular taxa can potentially employ one or more loci with higher phylogenetic resolution, for instance 16S–23S internal transcribed spacers (ITS) (e.g. van Gremberghe *et al*., 2011) or protein-encoding sequences (e.g. Whitaker *et al*., 2003).

A general problem in biogeography studies on both macro- and microorganisms is the confirmation of the absence of a studied taxon at a particular site or in a particular region. Cultivation-based investigations on microorganisms may easily overlook the presence of a certain taxon in samples because of the large diversity and high cell numbers of microbial communities. In order to lessen this problem, we studied in a first step the distribution of taxa by a cultivation-based approach, and tested in a second step for absence of taxa in certain regions by using environmental DNA samples and polymerase chain reaction (PCR)-driven detection methods, i.e. cultivation-independent methods. This combination of approaches and the fact that the studied organisms usually form persistent and well-detectable populations (Hahn *et al*.,;^2005^,^2012^) strongly decreased the probability of false negative detection results.

Here we study the biogeography of planktonic freshwater bacteria currently classified as Polynucleobacter necessarius subspecies *asymbioticus* (Hahn *et al*., 2009), which are also known as free-living PnecC bacteria (Hahn, 2003). This taxon represents a phylogenetically narrow group characterized by 16S rRNA gene sequence similarities of ≥ 99.2%. Ecologically, this taxon is characterized by an apparently ubiquitous presence in standing freshwater systems (Jezberová *et al*., 2010; Jezbera *et al*., 2012) and a cosmopolitan distribution (Hahn, 2003; Pearce *et al*., 2003; Crump *et al*., 2004; Hahn *et al*., 2009; Ghai *et al*., 2011). They contribute on average by 10% and 20% (extrapolated) to bacterioplankton cell numbers in Central European lentic freshwater systems (Jezberová *et al*., 2010; Jezbera *et al*., 2012) and in freshwater systems globally (Jezberová *et al*., 2010) respectively. Investigations employing phylogenetic markers with resolution beyond the resolution of 16S rRNA sequences suggested that ubiquity of the entire group in freshwater systems results from the presence of specialized lineages in systems differing in ecological conditions (Jezbera *et al*., 2011). Thus, the ecologically wide distribution of the subspecies is explained by an ecological radiation into differently specialized lineages, for instance adapted to different pH conditions. This explanation for the ubiquitous occurrence of the taxon in freshwater systems is summarized by the ubiquity-by-diversification hypothesis (Jezbera *et al*., 2011).

The study presented here is based on a globally assembled collection of *P.n.* ssp. *asymbioticus* strains currently consisting of 204 isolates from 130 different sites. The collection contains strains from all six continents and all major climatic zones. We searched for phylogeography patterns by analysing sequences of two phylogenetic markers. The most important phylogeographic patterns suggested by the analysis of cultivated strains were tested and confirmed by cultivation-independent investigations.

## Results

**Figure 1:**
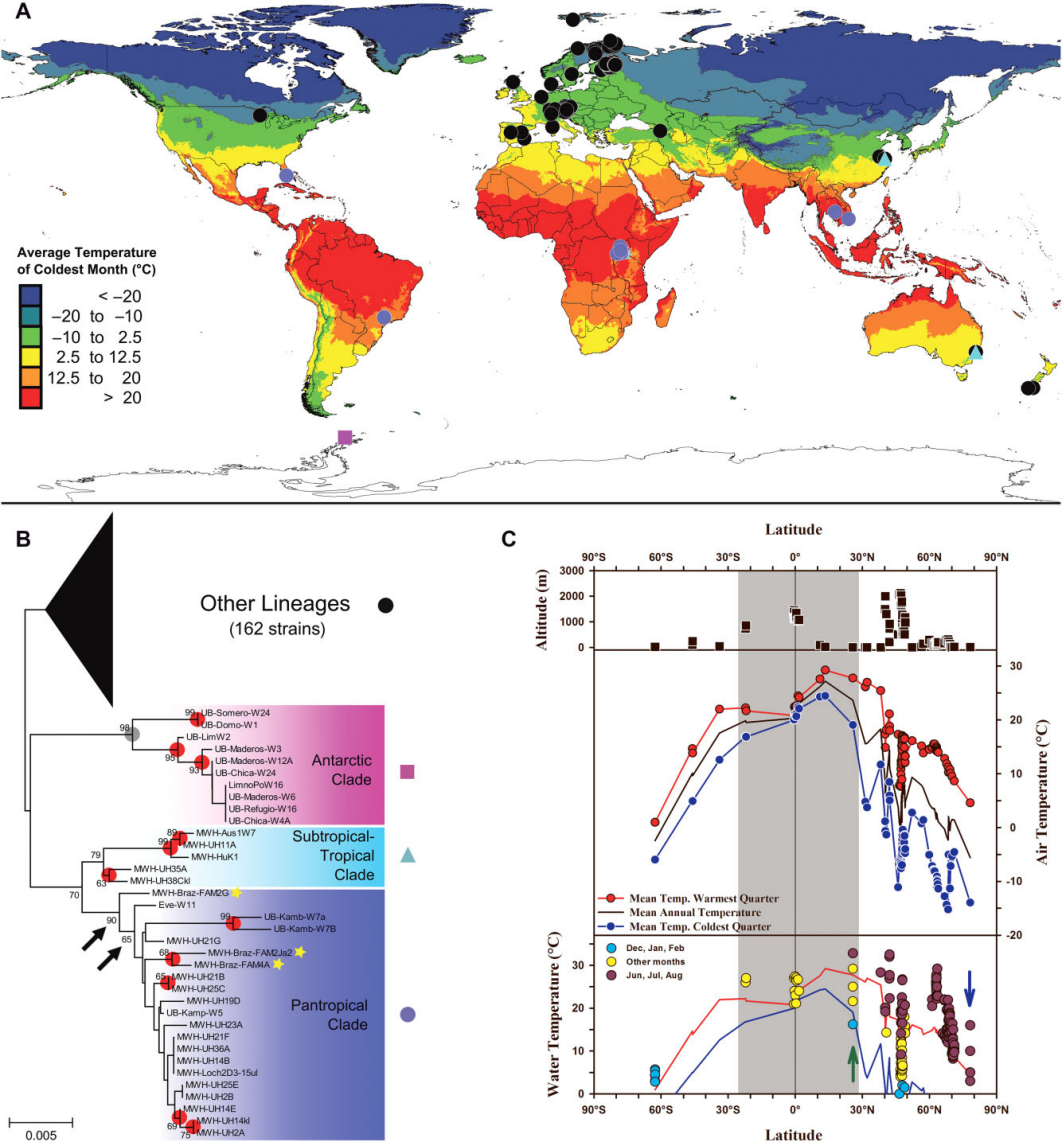
(A) Sites of origin of the cultivated 204 *P. necessarius* subspecies *asymbioticus* strains. Strains affiliated with the pantropical, tropical–subtropical and the Antarctic clade, as well as strains representing other lineages are depicted by different symbols (see B). Coloration of the map displays the average air temperature of the coldest month according to the WorldClim data set (Hijmans *et al*., 2005a). Note that the WorldClim data set does not provide data for Antarctica. Temperature data shown for the northern and southern hemisphere represent temperatures of the coldest months (January and July respectively). (B) Phylogenetic tree (NJ) of *P.n.* ssp. *asymbioticus* 16S–23S ITS sequences. The tree is based on the whole number of sequences but depicts mainly subgroups with major relevance to the current phylogeographic study. Nodes also present in trees based on partial glnA sequences are indicated by filled circles (red and grey dots indicate bootstrap support of > 60% in both trees and < 60% in at least one tree respectively). The clusters formed by the nodes indicated by arrows are also present in glnA trees; however, they lack the Brazilian strains (tagged by yellow stars). Complete tree of both markers are presented in Supporting Information Fig. S1. (C) Climatic conditions (air temperature) and water temperatures at sites of origin of the 204 *Polynucleobacter* strains. The top panel depicts the altitude of sites; the middle panel shows site-specific (filled circles) climate data provided by the WorldClim data set (data of 62–63°S were recorded on site, i.e. Byers Peninsula, Livingston Island) and the bottom panel shows site-specific water temperature data. Most of the latter data represent surface water temperatures of habitats of origin of the cultivated strains. For better illustration of ecological conditions some data of sampled habitats north of 60°N not represented by cultivated strains were added. Additionally, data recorded at station NP202 (US Geological Survey) located in the Everglades, Florida (25°N, green arrow) close to the site of origin of one *Polynucleobacter* strain affiliated with the pantropical clade, and data of a small Arctic lake (Zwoliñski *et al*., 2007, blue arrow) located on Svalbard near the site of origin of another *Polynucleobacter* strain are plotted in the bottom graph. Data of the Everglades site represent 95 and 75 percentile, mean temperature and 25 and 5 percentile (top to bottom) of a data set covering several years. For comparison, air temperature data presented in the middle panel are also shown in the bottom graph (red and blue lines). The gray area displayed throughout the three panels indicates the approximate latitudinal range limit of the pantropical clade.

The habitats from which the 204 *P.n.* ssp. *asymbioticus* strains (Supporting Information Table S1) were isolated represent an ecologically broad spectrum of lakes and ponds distributed over all six continents and all major climatic zones (Fig. 1). A minor fraction of strains were isolated from running waters (5 strains), a reservoir (2 strains) and puddles on forestry roads (3 strains). Besides large differences in size, these habitats also vary strongly in physico-chemical conditions. For instance, pH and conductivity values ranged from 3.5 to 9.0 and 4 to 10 010 μS cm^−1^ respectively. The latitudinal distribution of sites ranges from 78°N (Arctic region, Spitsbergen, Svalbard Islands, Norway) to 63°S (Antarctic Peninsula) (Fig. 1A). While the continent-specific origin of strains is strongly biased towards Eurasia (80% of strains) a more even climate-specific origin of strains can be noted (Table 1). Polar/alpine sites were located in the Arctic (i.e. north of 10°C July isotherm; 5 habitats, 7 strains), the maritime Antarctic (6 habitats, 10 strains) and at high altitudes in the European Alps (13 habitats, 20 strains).

**Table 1:** Climatic and geographic origin of *Polynucleobacter necessarius* ssp. *asymbioticus* strains

Köppen–Geiger climate (1st category)	Number of strains						
	Total	Eurasia (Europe)^f^	Africa	North America	South America	Oceania	Antarctica
A, tropical^a^	23	4 (0)	18	1	–	–	–
C, temperate^b^	64	55 (49)	–	–	3	6	–
D, continental^c^	80	78 (78)	–	2	–	–	–
E, polar/alpine^d^	37	27^e^ (27^e^)	–	–	–	–	10
Total	204	164 (154)	18	3	3	6	10

a All 12 months of the year have average temperatures of 18°C or higher.

b Average temperature above 10°C in warmest months, and coldest month average between −3°C and 18°C.

c Average temperature above 10°C in their warmest months, and coldest month average below −3°C.

d Average temperatures below 10°C in all 12 months of the year.

e Data in brackets indicate the number of strain from Europe.

f Twenty alpine strains (Alps), seven Arctic strains.

Strains were climatically classified by using the first category of the Köppen–Geiger climate classification system (Strahler and Strahler, 2005).

The genetic markers used in the current investigation differed in sequence diversity. The nucleotide diversity of the 16S–23S ITS (543 alignment positions) and glnA sequences (603 bp) were 0.022 and 0.114 respectively. While the average similarity of ITS sequences was 97.5% (range 92.8–100%), the partial glnA sequences showed average sequence similarity of 88.7% (range 82.1–100%) and 97.0% (range 92.0–100%) on the nucleotide and amino acid levels respectively.

### Global phylogeography of *P.n.* ssp. *asymbioticus*

Mantel tests were performed to search for a potential isolation by distance. All strains were included in this analyses, although (despite of the fact that) nine of the ten Antarctic strains were isolated at incubation temperatures of 15°C, and all other at room temperature (>20°C). A cultivation bias due to the different isolation temperatures cannot be ruled out; however, Antarctic strains, which were isolated at room temperature and 15°C, respectively, shared identical ITS and glnA sequences. Furthermore, cultivation-independent investigation with *Polynucleobacter*-specific primers (Hahn *et al*., 2005) on the *Polynucleobacter* diversity in one of the investigated Antarctic lakes did not reveal the presence of any *Polynucleobacter* lineage missed by the cultivation approach (data not shown). Both phylogenetic markers analysed indicated a significant correlation between genetic and geographic distance (Table 2), which suggests isolation-by-distance pattern on a global scale. Significant but weak correlations between climate parameters (air temperature) and genetic distance of the glnA marker were also revealed, whereas the ITS marker showed no significant correlations with these climate parameters. By contrast, no significant correlation between differences in pH values of habitats from which strains were isolated and the genetic distance between strains could be observed for any of the phylogenetic markers assayed.

**Table 2:** Results of Mantel tests with data on pairwise genetic distance (Y) of isolates determined either for their 16S–23S ITS sequences or their partial glnA gene sequences against pairwise distance/differences of various parameters (X)

	Geographic distance	Annual temperature	Warmest month	Coldest month	Warmest quarter	Coldest quarter	pH
glnA (nt, 202 Sequences)							
Correlation coefficient (rY)	0.210	0.098	0.126	0.079	0.115	0.078	−0.027
Determination of Y by X (%)^a^	4.4	1.0	1.6	0.6	1.3	0.6	–
P (rY_rand_ ≥ rY_obs_)	<0.0001	0.0001	<0.0001	0.0016	<0.0001	0.0015	0.86
ITS (nt, 204 sequences)							
Correlation coefficient (rY)	0.276	−0.035	−0.004	−0.046	−0.011	−0.049	−0.082
Determination of Y by X (%)^a^	7.6	–	–	–	–	–	–
P (rY_rand_ ≥ rY_obs_)	<<0.0001	0.90	0.54	0.95	0.65	0.97	1.00

a *P* < 0.01.

These parameters included geographic distance of sites of origin of the strains, pH measured on site and several climate parameters predicted by a climate model for the sites of origin of the strains. Note that the climate data refer to air temperatures, which represent only rough proxies for thermal conditions in aquatic systems.

A closer look at the reconstructed phylogeny of strains revealed phylogeographic patterns of restricted geographic ranges of subgroups (Fig. 1A and B). One clade representing 10% of strains is exclusively represented by organisms originating from habitats at sites with a warm, mainly tropical climate (temperatures above 18°C in all 12 months; Peel *et al*., 2007) or almost tropical climate (three strains from two sites in Brazil with minimum monthly mean temperatures of 17°C). As the sites of origin of these 21 strains are spread within tropical areas from South and North America, Asia and Africa, the clade formed by these organisms was named pantropical clade (Fig. 1B). Mantel tests on correlations of the genetic distance between strains of this clade and geographic distance, or selected environmental or climatic parameters, revealed a pronounced correlation with geographic distance (r = 0.42, *P* < 0.01, and r = 0.39, *P* < 0.01 for ITS and glnA markers respectively) but no significant correlation between genetic distance and any of the tested environmental or climatic parameters (data not shown). Thus, compared with the entire set of strains, a much stronger spatial influence on genetic divergence was suggested for the pantropical clade.

### Testing of range restrictions of the pantropical clade

The pantropical clade is almost identical with the previously characterized group F13n (Jezbera *et al*., 2011), which was defined based on the presence of a diagnostic oligonucleotide sequence in the 16S–23S ITS sequences of member strains. Only one strain of the pantropical clade lacks this diagnostic sequence (Supporting Information Fig. S1), thus 95% of the known clade members could be detected by a previously developed reverse line blot hybridization (RLBH) probe (Jezbera *et al*., 2011). Importantly, previous investigation of 121 samples from non-tropical habitats in Europe by RLBH resulted in a complete lack of detection of the pantropical clade, while other *Polynucleobacter* groups were frequently detected in these samples (Jezbera *et al*., 2011 and Table 3). By contrast, investigation of environmental DNA extracted from 30 freshwater habitats in tropical Uganda (Supporting Information Table S2) by RLBH resulted in 63% of habitats being positive for the pantropical clade (82% of habitats yielding PCR products were positive). Presence of strains affiliated with this clade was also confirmed by an analysis of a metagenomic data set representing microbial communities of the Amazon River (Ghai *et al*., 2011). Our analysis revealed that this metagenomic data set contains a couple of glnA sequences clustering within the pantropical clade (data not shown).

**Table 3:** Detection of the pantropical, the F1 and the Antarctic clade in various regions by cultivation and cultivation-independent methods

Taxon	Origin of Samples	Method	Total number of investigated samples or strains	Positive samples or strains (#)	Positive samples or strains (%)	Reference
Pantropical clade	Europe	RLBH (probe F13n), env. DNA	121	0	0,0	Jezbera *et al*., 2011
Pantropical clade	Uganda	RLBH (probe F13n), env. DNA	30 (22)^b^	18	60.0 (81.8)^b^	This study
Pantropical clade	Outside tropics	Cultivation, strains	178	0	0,0	This study
Pantropical clade	Tropics	Cultivation, strains	26	21	80,8	This study
Antarctic clade	Outside Antarctica	Cultivation, strains	194	0	0,0	This study
Antarctic clade	Antarctica	Cultivation, strains	10	10	100,0	This study
Antarctic clade	Europe	PCR, env. DNA	57	0	0,0	This study
Antarctic clade	Antarctica	PCR, env. DNA	16	12	75,0	This study
Clade F1^a^	Europe	RLBH (probe F1), env. DNA	121	0	0,0	Jezbera *et al*., 2011
Clade F1^a^	Uganda	RLBH (probe F1), env. DNA	30 (22)^b^	16	53.3 (72.7)^b^	This study

a Clade F1 is only represented by two strain, one isolated from a tropical site and one isolated from a subtropical site (see Suppl. Materials Fig. S1).

b Only 22 of 30 samples resulted in PCR products suitable for analysis by RLBH.

RLBH, reverse line blot hybridization; env. DNA, environmental DNA; #, number.

**Figure 2:**
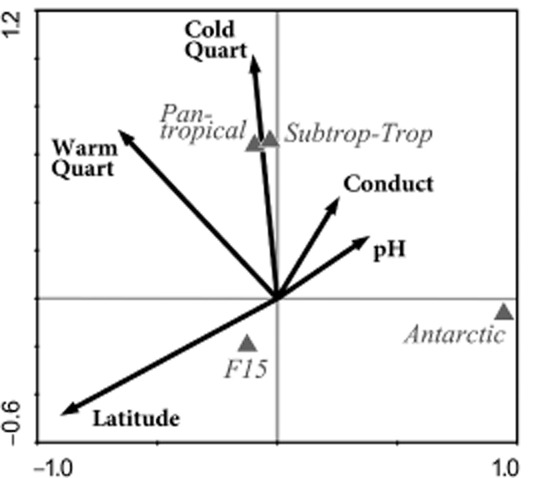
Canonical correspondence analysis (CCA) of the relationship of particular clades (triangles) to environmental and climatic conditions (arrows) characterizing the habitat from which the clade members were isolated. This two-dimensional model explains 63% of the observed variability. Only clades that showed significant correlations in this model are depicted. Conduct, conductivity (μS per centimetre); WarmQuart, mean air temperature of warmest quarter; ColdQuart, mean air temperature of coldest quarter. For data on clade F15 see Suppl. Materials Figs S1 and S2.

**Figure 3:**
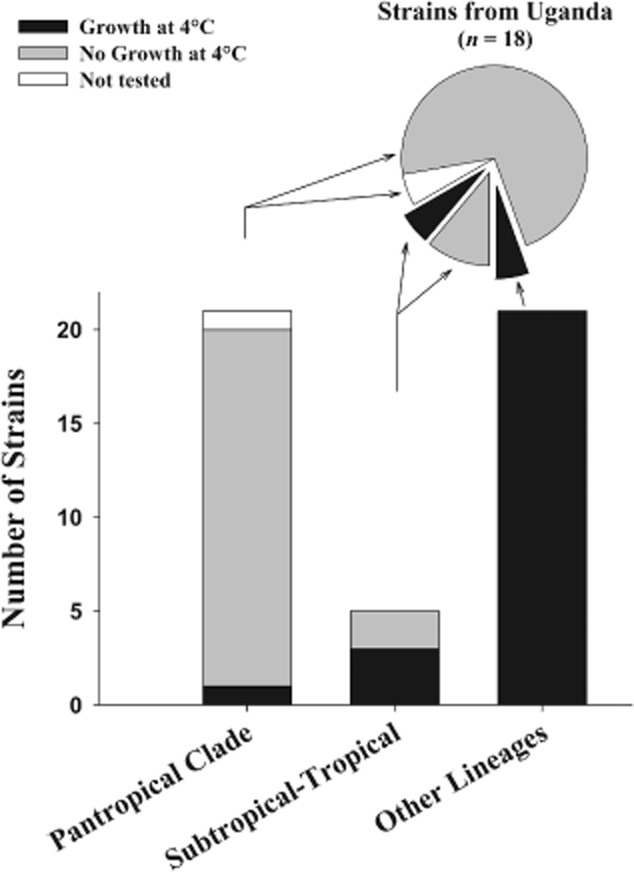
Ability of *Polynucleobacter necessarius* strains affiliated with the pantropical, the subtropical–tropical and other lineages to grow at 4°C on NSY medium. Positive controls incubated at room temperature were positive for all tested strains. The bar chart shows the results for all 42 tested strains. The pie chart depicts the results of the 18 strains (included in the bar chart) obtained from sites in Uganda (Central Africa). Sixteen, three and one of the Ugandan strains are affiliated with the pantropical clade, the tropical–subtropical clade and another lineage respectively.

Canonical correspondence analysis (CCA) indicated a significant influence of climate proxies (air temperature) and geographic (latitude) parameters on distribution of the pantropical clade (Fig. 2) compared with the other clades. Both of which suggest the influence of thermal conditions on the range of this taxon. As suggested by climate data, as well as by the available data on water temperatures in habitats of origin of the *Polynucleobacter* strains, freshwater systems inhabited by the pantropical clade differ from the other habitats mainly in the absence of low winter temperatures and weaker seasonality (Fig. 1C). Even in several habitats northern than 60° latitude surface water temperatures > 25°C were measured, which are comparable with typical surface temperatures of large tropical lakes such as Tanganyika or Malawi (World Lake Database, http://wldb.ilec.or.jp/LakeDB2/). By contrast, typical winter temperatures of non-tropical lakes, i.e. < 10°C, cannot be expected in lowland tropical freshwater systems. We tested if this basic difference between tropical and non-tropical habitats is reflected by the thermal adaptation of *Polynucleobacter* strains (Fig. 3). Only one of the 20 tested pantropical clade strains grew at 4°C, but showed only weak growth (colony size) in comparison with the control culture. In contrast, all 20 tested non-tropical strains affiliated with diverse lineages grew well at this low temperature, while only three out of five strains of the subtropical–tropical clade were able to grow.

### Endemicity of the Antarctic clade?

In total, 37 strains were isolated from cold environments, i.e. polar and alpine lakes and ponds (Table 1). Strains isolated from southern (Antarctic) and northern hemisphere (Arctic and alpine) habitats clustered separately in all phylogenetic reconstructions performed. While the Antarctic strains formed one (ITS sequences, GlnA AA sequences) or two monophyletic clusters (glnA nt sequences) (Fig. 1B), most of the Arctic and alpine strains appeared to be more closely related to strains obtained from temperate sites.

We intended to search for the Antarctic genotypes in the tropics and the northern hemisphere. Due to the lack of a diagnostic ITS sequence, a RLBH probe suitable for detecting the Antarctic clade could not be developed. Instead, a primer pair for specific detection of the larger branch of the Antarctic clade (Supporting Information Fig. S1) was developed and applied in a nested PCR approach to 16 environmental DNA samples from Antarctic lakes on Livingston Island and at Hope Bay, and 57 samples from lakes and ponds in Uganda (13 habitats) and Europe (44 habitats). The European sites included 10 subalpine and 7 alpine habitats, all located in the Austrian Alps. In almost all cases, nested PCR with environmental DNA of non-Antarctic habitats yielded in the first PCR step a PCR product. This confirmed the presence of *Polynucleobacter* bacteria. In the second PCR step, however, only one non-Antarctic sample resulted in a PCR product. Sequencing of this sole PCR product resulted in a sequence clustering in phylogenetic trees outside of the Antarctic clade. Consequently, this PCR product was not scored as detection of the clade (Table 3). Thirteen of the 16 samples of Antarctic lakes resulted in detection of the Antarctic clade either by cultivation or nested PCR or both (Supporting Information Table S3).

## Discussion

Previous studies on diversity and environmental distribution of *Polynucleobacter* bacteria focused on sites in Central Europe (Jezbera *et al*., 2011). Here we enlarged the focus to a global scope and revealed a phylogeographic pattern influenced by geographic distance and climate. Importantly, pH representing the most important environmental parameter strongly influencing *Polynucleobacter* community composition on a regional scale (Jezbera *et al*., 2011) was not found to influence genetic distance between strains on a global scale (Table 2). Instead, climate-related parameters and isolation-by-distance mechanisms are suggested to structure *Polynucleobacter* communities on this scale.

The used climate data are only weak but are the only available proxies for year-round thermal conditions in the freshwater systems inhabited by the isolated bacterial strains. During warmer seasons, surface water temperatures of lakes and ponds are usually above average air temperatures (Fig. 1C) due to the heat storage capacity of standing water bodies. At sites with winterly average air temperature below 0°C bacteria and other organisms do not experience growth conditions below freezing point. Vertical differences of water temperatures in stratified lakes are only a minor point here, because almost all (one exception) bacterial strains contained in the culture collection were obtained from surface waters. Due to the limitations of climate data as proxies for thermal conditions in standing aquatic systems, the observed weak correlation between glnA genetic data and climate data may underestimate the influence of thermal conditions on the global structuring of the *Polynucleobacter* population. Lack of correlation with ITS data could, in addition, have been influenced by the lower genetic diversity of this marker (Hahn *et al*., 2012) and by the presence of hypervariable regions potentially masking phylogenetic signals due to extensive homoplasy (van Gremberghe *et al*., 2011).

### Do range restrictions of subgroups really exist?

Of course all the results by cultivation and molecular detection experiments (Table 3) do not provide evidence for complete absence of taxa in the entire tropical or non-tropical zones respectively. Complete absence is simply a non-testable hypothesis. Even ultra-deep sequencing of environmental DNA cannot prove the absence of a specific taxon in a certain area. Another potential reason for overlooking microorganism in a certain habitat is sampling of the system during an inappropriate period characterized by only low numbers of the taxon of interest. This is especially a problem in bloom-forming taxa, while *Polynucleobacter* bacteria rather tend to establish persistent populations fluctuating in numbers much less than typical bloom-forming microorganisms (Hahn *et al*.,;^2005^,^2012^; Wu and Hahn,,2006a,2006b). Although, as stated above, a perfect prove of complete absence of a certain taxon in a certain natural system is basically impossible, the observed patterns of detection/no detection obtained by two independent methods are at least for some subgroups very strong (Table 3). It is important to note that a previous study demonstrated that cultivation and cultivation-independent (cloning and sequencing) investigations on the *Polynucleobacter* population of a humic pond revealed the same diversity of 16S–23S ITS genotypes and also suggested a low genetic diversity of *Polynucleobacter* populations (Hahn and Pöckl, 2005; Hahn *et al*., 2005). Thus, it is not expected that cultivation-based investigation results in a strongly biased picture of the *Polynucleobacter* diversity.

The phenomenon of restricted ranges seems not to be limited to the pantropical and the Antarctic subgroup. The currently available distribution data (based on both cultivation and RLBH) also suggest for some other *Polynucleobacter* clades geographic range restrictions. This includes the subtropical–tropical clade (Fig. 1), which is partially represented by the previously defined group F2 (Jezbera *et al*., 2011), clade F1 (Table 3; Jezbera *et al*., 2011), clade F10 (Jezbera *et al*., 2011) and clade F15 (Jezbera *et al*., 2011), which is represented by 10% of the isolated strains (Table 4, Supporting Information Fig. S1). For groups F1, F10 and F15, suitable RLBH probes were available for testing presence/absence in Central European and African habitats located in Uganda. Similar to the pantropical clade, group F1 could not be detected in Europe, but it was detected in several Ugandan habitats (Table 3). Whereas for the other two groups, an opposite trend was observed. In one case very weak (compared with detections in European samples), most likely false positive, detections in Ugandan samples were noted, though (Supporting Information Table S2). Further investigations are required for confirming these trends, however, these observations suggest that range restrictions are not exceptional among subgroups of *Polynucleobacter* bacteria.

**Table 4:** Genetic and ecological characteristics of three putative endemic *Polynucleobacter* taxa. All data are based on isolated representatives

Taxon	Number of strains	Number of haplotypes		Minimum similarity (%)		Nucleotide diversity Pi		pH range	Maximum distance
		ITS	glnA	ITS	glnA	ITS	glnA		(km)
Pantropical clade	21	15	19	97.3	86.1	0.01005	0.07798	5.7–8.2	17234
F15	20	5	8	99.2	98.8	0.00272	0.00199	4.2–7.2	3264
Antarctic clade	10	5	5	97.9	85.2	0.00881	0.0621	6.8–8.5	10 (222)^a^

a Maximum distance of sites of origin of cultivated strains is 10 km, maximum distance between sites of detection is 222 km.

Maximum distance and pH range refer to habitats from which strains affiliated with the taxa were obtained. See Supporting Information Fig. S2 for estimated range limits of each of the three taxa.

Based on the current data, it is not possible to establish detailed estimations on the range boarders of subgroups. The geographic pattern of detection of the pantropical clade, the correlation of the distribution with climatic data and the revealed inability of strains to grow at low temperatures suggest that this clade is more or less restricted to the tropical climatic zone. However, whether and how far the range extends into the subtropical zones of both hemispheres is currently unknown (Supporting Information Fig. S2).

The Antarctic clade was observed to be widely distributed in lakes on Byers Peninsula (Camacho, 2006; Toro *et al*., 2007; Villaescusa *et al*.,;^2010^,^2013^) and at Hope Bay, 222 km distant (Supporting Information Table S3), while there was no hint of the presence of this clade in European and African lakes and ponds. Consequently, the Antarctic clade is currently known exclusively from several freshwater lakes located at the Antarctic Peninsula (Supporting Information Fig. S2). Whether the clade is restricted to Antarctic freshwater habitats or it is also present on other continents, is currently not known. The presence of *Polynucleobacter* in other freshwater lakes of maritime Antarctica was demonstrated previously (Pearce *et al*., 2003; Villaescusa *et al*., 2013), but these detections were exclusively based on 16S rRNA sequences as opposed to the analysis of 16S–23S ITS, and so whether those *Polynucleobacter* bacteria belong to the Antarctic clade cannot be analysed. Currently, we cannot exclude that at least alpine Andean lakes may represent habitats outside Antarctica potentially colonized by the cold-adapted Antarctic clade (Supporting Information Fig. S2).

### Thermal adaptation of strains

The pantropical subgroup showed thermal adaptations different from strains isolated from habitats located in the temperate climatic zone (Fig. 3). Tropical strains are characterized by a lack or very weak growth at low temperatures, which fits well to the thermal conditions in their home habitats. Climate-linked differences in thermal adaptation of closely related strains of planktonic freshwater bacteria affiliated with another phylum were demonstrated previously (Hahn and Pöckl, 2005). This previous study compared optimal and maximum growth temperatures of actinobacterial strains isolated from habitats located in three climatic zones. The seven investigated strains shared identical 16S rRNA sequences and the 16S–23S ITS sequences possessed sequence similarities in the range of 99.0–100%. Thus, sequence diversity of both markers was smaller than sequence diversity revealed for the *Polynucleobacter* strains investigated in the current study. However, even these closely related strains of *Actinobacteria* showed pronounced differences in thermal adaptations, which reflected differences in thermal characteristics of their home habitats.

### Intercontinental distribution of the pantropical clade

The range of the pantropical clade was found to be longitudinally unlimited within the tropical climatic zone (Fig. 1A). This could either be explained by a historical distribution already present before the break-up of the supercontinent Pangaea about 150–140 Ma before present or alternatively by later colonization of parts of the distribution area by natural or anthropogenically assisted intercontinental dispersal. Such successful intercontinental dispersal was demonstrated for spore-forming bacteria (Gorbushina *et al*., 2007). Interestingly, *Polynucleobacter* bacteria were detected in the troposphere (DeLeon-Rodriguez *et al*., 2013), but whether *Polynucleobacter* bacteria, which are unable to form spores, are transported over large distances without loss of viability, remains unknown. If gene flow between continents was absent or limited, separation of palaeotropical and neotropical subgroups of the clade should have resulted in genetic divergence following the break-up of Pangaea. By assuming isolation over a period of 140 Ma and an assumed divergence rate of the 16S rRNA genes of 0.00625–0.0125% per Ma, which represent conservative estimates of 25–50% of the slowest rate estimated for obligately endosymbiotic bacteria (Kuo and Ochman, 2009), sequence divergence within the pantropical clade of 0.9–1.8% could be expected. The lower divergence value is similar to the maximum divergence (0.8%) observed for the entire *P.n.* ssp. *asymbioticus* group (64 16S rRNA sequences ≥ 1477 bp). However, the maximum palaeo-neotropical divergence of almost full-length 16S rRNA sequences affiliated with the pantropical clade was only 0.2%, while a palaeotropical (Asia) and two neotropical strains (South America) even shared identical 16S rRNA sequences. Furthermore, in contrast to other bacterial taxa (Bahl *et al*., 2011), the phylogenetic reconstructions with both markers revealed no phylogenetic structure separating palaeo- and neotropical strains (Fig. 1B). Altogether, these findings suggest successful intercontinental dispersal of pantropical clade strains. The observed distance-decay pattern hints at rare but natural intercontinental dispersal events too weak to balance divergence between distantly located populations. On the other hand, this pattern argues against a recent pantropical range expansion driven by anthropogenic activities, such as intercontinental transport of freshwater as ballast water (Seiden *et al*., 2010).

### Phylogenetic resolution and intra-taxon ecological diversification

As stressed previously, phylogenetic resolution of methods applied in phylogeographic investigations on microorganisms is the crucial factor for depths of analysis (Bass and Boenigk, 2011; Hanson *et al*., 2012). While the 16S rRNA gene of *Polynucleobacter* bacteria lacks almost any intraspecific phylogeographic signal (Hahn, 2003), the two markers employed in this study revealed a deep phylogeographic structure in *P.n.* ssp. *asymbioticus*. The presented analysis suggests that this structure results from both environmental selection of ecotypes and restrictions in gene flow between distant sites. Weighting of the proportional impact of these two mechanisms on the phylogeographic structuring will require larger data sets on ecologically more homogenous subgroups.

The analysed strain collection is currently geographically biased due to overrepresentation of strains obtained from European sites. This geographic bias and the small number of phylogenetic markers included in the study are potentially limiting the potential for revealing intercontinental structuring of *Polynucleobacter* communities. For instance, genetic differentiation between North American and European populations potentially caused by geographic isolation could not be revealed due to the lack of a suitable number of strains originating from North America. Future isolation efforts should focus on sites outside of Europe. Furthermore, efforts for establishment of a multilocus sequence typing scheme for *Polynucleobacter* bacteria are required. Both improvements will enable analysis of biogeographic structures with an increased resolution.

Apart from geographic reasons, the environmental filtering of ecotypes seems to take place at two different levels. On a regional level, climatic conditions seem to act as a filter, sorting out ecotypes with unsuitable thermal adaptation (Fig. 3). At a local level, habitat-specific environmental conditions such as pH, conductivity, oxygen concentration, and quantity and quality of dissolved organic carbon further select for suitably adapted genotypes (Jezbera *et al*., 2011). This two-level environmental filtering results in a patchy phylogeographic structure, which is more complex than revealed so far.

## Conclusions

This study revealed a dimension of microbial diversity, which cannot be discovered by current ultra-deep sequencing projects employing next generation sequencing of 16S rRNA markers. Linking of genetic diversity data and phenotypic data suggested a phylogeographic structuring of the *P.n.* ssp. *asymbioticus* population, which is at least partially shaped by adaptation to different thermal niches. Future studies have to reveal the extent of the ecological differences between subgroups involved in this phylogeographic structuring, as well as the contribution of restrictions in gene flow to the revealed patterns.

## Experimental procedures

### Samples

The study is based on two different kinds of samples, i.e. bacterial strains representing the investigated taxon and environmental DNA directly extracted from water samples. The strains were isolated as pure cultures from various freshwater systems by using the acclimatization method (Hahn *et al*., 2004). The established culture collection consists of 204 *Polynucleobacter necessarius* subspecies *asymbioticus* (Hahn *et al*., 2009) strains. Samples of environmental DNA were obtained from many freshwater systems by sampling surface waters (up to 0.5 m depth), harvesting of planktonic organisms on 0.2 μm filters and subsequent DNA extraction as described previously (Jezbera *et al*., 2011).

### Sequencing of genetic markers and phylogenetic analysis

16S–23S ITS and partial glutamine synthetase gene sequences (glnA) were amplified from gDNA of pure cultures of strains, sequenced and phylogenetically analysed as described previously (Jezbera *et al*., 2011). Both markers represent single copy genes/sequences in *Polynucleobacter* genomes (Hahn *et al*., 2012).

### Detection of taxa by RLBH

16S–23S ITS sequences of *P.n.* ssp. *asymbioticus* were amplified from environmental DNA samples by using taxon-specific primers, as described previously (Jezbera *et al*., 2011). The obtained mixtures of amplified ITS sequences were investigated for the presence/absence of certain *Polynucleobacter* subgroups by RLBH by using a set of thirteen *Polynucleobacter*-subgroup-specific hybridization probes (Jezbera *et al*., 2011).

### Detection of the Antarctic clade by PCR

A primer pair for specific detection of Antarctic *Polynucleobacter* genotypes in environmental DNA samples was developed. The primers Limnop-glnA-F (5′-CATCATTAAGCATGCGAAAGCAC-3′) and Limnop-glnA-R (5′-CCATCATCAATGCAGCAAAGCAA-3′) target the glnA genes of 8 of the 10 obtained Antarctic *Polynucleobacter* strains. PCR conditions were optimized by using gDNA extracted from target strains. A nested PCR approach was applied to environmental samples in order to increase the sensitivity of detection of Antarctic genotypes. The *Polynucleobacter*-specific primers glnA1212F and glnA1895R (Jezbera *et al*., 2011) were used in a first step for amplifying partial glnA sequences. PCR products were subjected to a second PCR with the new primers Limnop-glnA-F and Limnop-glnA-R.

### Thermal adaptation of cultivated strains

The capability of selected strains to grow at low temperatures was tested by dropping 10 μl of culture grown in liquid NSY medium (Hahn *et al*., 2004) on NSY agar plates and subsequently incubating them (triplicates for each strain) at 4°C for up to 15 weeks. Positive controls were incubated at room temperature (> 23°C). Experiments resulting in lack of growth at 4°C were repeated.

### Climate data

Climate data were used as proxies of thermal conditions of aquatic systems from which *Polynucleobacter* strains were isolated. Site-specific climate data were obtained from the WorldClim data set (v. 1.3, with a resolution of 2.5 min) (Hijmans *et al*., 2005a), covering the global land areas except Antarctica, using the diva-gis software (Hijmans *et al*., 2005b). This included data on annual mean air temperature, mean maximum air temperature of the warmest month, mean minimum air temperature of the coldest month, mean air temperature of the warmest quarter and mean air temperature of the coldest quarter. Climate data at the site of origin of the Antarctic strains (Byers Peninsula, Livingston Island, South Shetlands Islands, and Hope Bay, Maritime Antarctica,) were recorded on location (Villaescusa *et al*., 2010; Schiaffino *et al*., 2011; Bañon *et al*., 2013).

### Statistical analyses

Using Mantel tests, isolation by distance was investigated as the correlation between matrices of pairwise genetic distances of strains and matrices of pairwise geographic distances of sites of origin of strains. In the same way, correlations of genetic distance and differences in climatic or pH conditions between sites of origin of strains were searched. Pairwise genetic distances between strains were calculated for both markers (nt sequences) by using the Tamura 3-parameter substitution model with non-uniformity of evolutionary rates among sites and by assuming that a certain fraction of sites are evolutionarily invariable (T92 + G + I substitution model) by using the mega software, version 5 (Tamura *et al*., 2011). Pairwise geographic distances (great-circle distance) were calculated with the Geographic Distance Matrix Generator (Peter Ersts, American Museum of Natural History, Center for Biodiversity and Conservation, http://biodiversityinformatics.amnh.org/open_source/gdmg) from geographic coordinates. Using the arlequin software (Excoffier *et al*., 2005), correlations between matrices were analysed by Mantel tests with 100 000 permutations.

Multivariable analyses were performed using the canoco software (Ter Braak and Šmilauer, 1998). CCA was carried out with centring and standardization by species norm. Forward selection was used to choose significant (*P* < 0.05) explanatory (environmental) variables by a Monte Carlo permutation test with 999 unrestricted permutations.

### Nucleotide sequences

Accession numbers are provided in Supporting Information Table S1.

## Supporting Information

Fig S1 Complete phylogenetic trees (NJ) of the 16S–23S ITS and the glnA sequences (compare Fig. 1B). Subgroups mentioned in the paper are highlighted by different colours. Strains matching to the RLBH probe F13n targeting the Pantropical clade (Jezbera *et al*., 2011) and strains matching to PCR primers used for targeting the major branch of the Antarctic clade (this study) are indicated by brackets.

Fig S2 Putative range limits of the Pantropical (red), the Antarctic (blue) and the F15 clade (yellow). Range limits deduced from detections by cultivation and cultivation-independent methods are shown as solid lines of a dot (Antarctic clade, region represented by Byers Peninsula and Hope Bay). Hypothetical range limits are shown as dashed lines.

Table S1 *Polynucleobacter* strains, geographic origin and Genbank accession numbers of 16S–23S ITS and glnA sequences.

Table S2 Detection of *Polynucleobacter necessarius* ssp. *asymbioticus* subgroups by reverse line blot hybridization (RLBH) in habitats located in Uganda.

Table S3 Antarctic lakes investigated for the presence of the Antarctic clade.
