# Supplementary material for: Global phylogeography of pelagic Polynucleobacter bacteria: Restricted geographic distribution of subgroups, isolation by distance and influence of climate
Source: Environ Microbiol. 2014 Jul 15;17(3):829–40. doi: 10.1111/1462-2920.12532 (PMC4361717; doi:10.1111/1462-2920.12532)

Figure S1. Complete phylogenetic trees (NJ) of the 16S-23S ITS and the glnA sequences (compare Fig. 1b). Subgroups mentioned in the paper are highlighted by different colours. Strains matching the RLBH probe F13n (Jezbera et al., 2011) targeting the Pantropical clade, and strains matching the PCR primers used for targeting the major branch of the Antarctic clade (this study) are indicated by brackets.

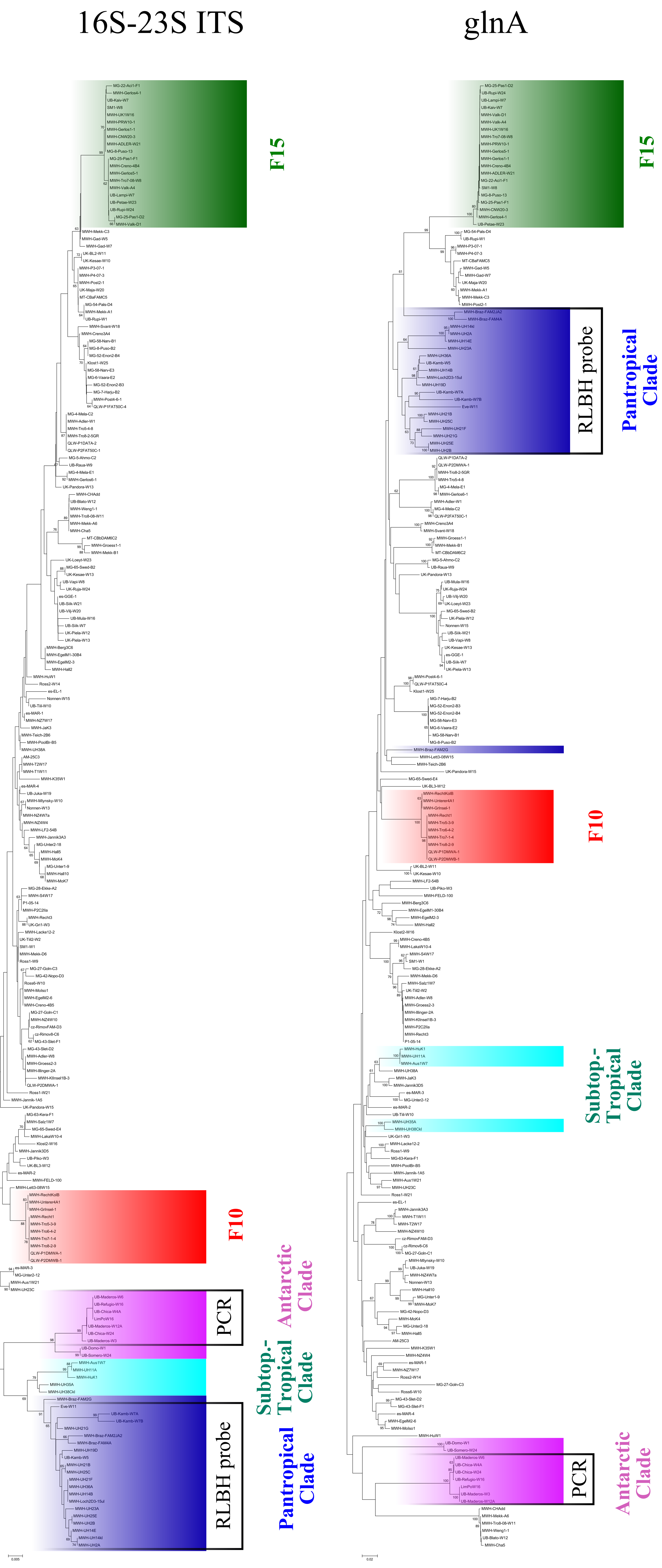

Supplement: Fig S1 — Complete phylogenetic trees (NJ) of the 16S–23S ITS and the glnA sequences (compare Fig. 1B). Subgroups mentioned in the paper are highlighted by different colours. Strains matching to the RLBH probe F13n targeting the Pantropical clade (Jezbera et al., 2011) and strains matching to PCR primers used for targeting the major branch of the Antarctic clade (this study) are indicated by brackets. [file emi0017-0829-sd1.pdf]
