# Supplementary material for: Global phylogeography of pelagic Polynucleobacter bacteria: Restricted geographic distribution of subgroups, isolation by distance and influence of climate
Source: Environ Microbiol. 2014 Jul 15;17(3):829–40. doi: 10.1111/1462-2920.12532 (PMC4361717; doi:10.1111/1462-2920.12532)

Figure S2. Putative range limits of the Pantropical (red), the Antarctic (blue), and the F15 clade (yellow). Range limits deduced from detections by cultivation and cultivation-independent methods are shown as solid lines (Pantropical and F15 clade) or a dot (Antarctic clade, region represented by Byers Peninsula and Hope Bay). Hypothetical range limits are shown as dashed lines.

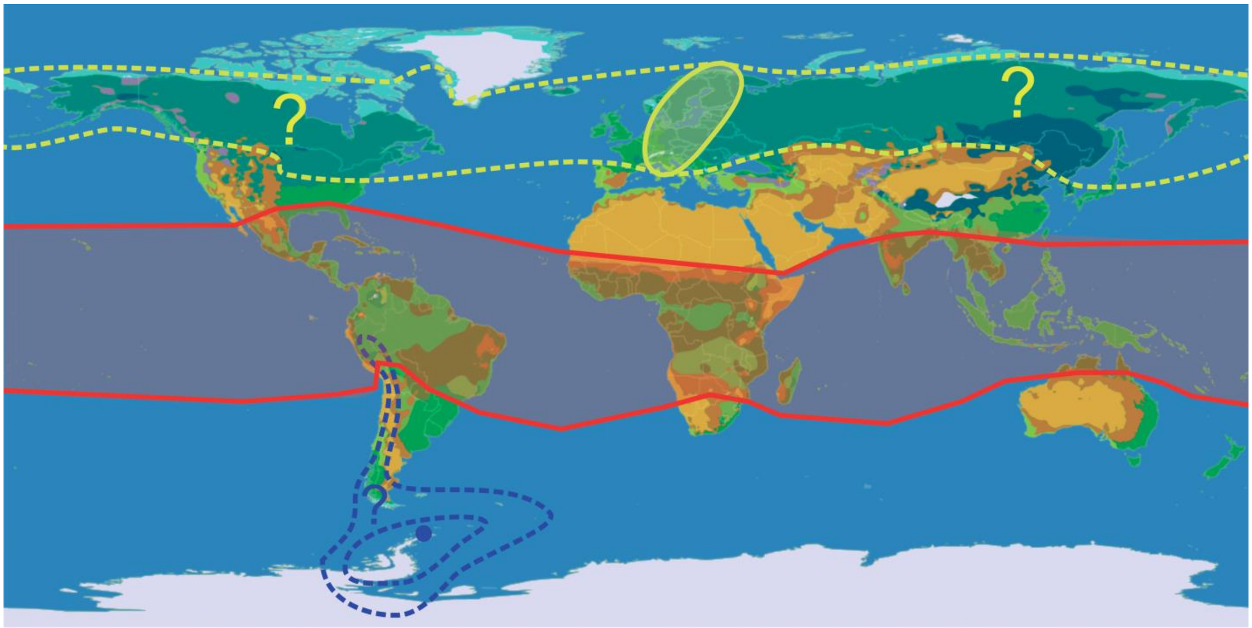

Supplement: Fig S2 — Putative range limits of the Pantropical (red), the Antarctic (blue) and the F15 clade (yellow). Range limits deduced from detections by cultivation and cultivation-independent methods are shown as solid lines of a dot (Antarctic clade, region represented by Byers Peninsula and Hope Bay). Hypothetical range limits are shown as dashed lines. [file emi0017-0829-sd2.pdf]
