# Supplementary material for: Global phylogeography of pelagic Polynucleobacter bacteria: Restricted geographic distribution of subgroups, isolation by distance and influence of climate
Source: Environ Microbiol. 2014 Jul 15;17(3):829–40. doi: 10.1111/1462-2920.12532 (PMC4361717; doi:10.1111/1462-2920.12532)
Supplement: Table S1 — Polynucleobacter strains, geographic origin and Genbank accession numbers of 16S–23S ITS and glnA sequences. [file emi0017-0829-sd3.pdf]

Table S1. *Polynucleobacter* strains, geographic origin, and Genbank accession numbers of 16S-23S ITS and glnA sequences.

Strain	Habitat	Latitude	Longitude	16S-23S ITS	glnA
MWH-MoIso1	Lake Mondsee	47.82975	13.37609	AJ550671	FN823082
MWH-MoK7	Lake Mondsee	47.82975	13.37609	AJ550673	FN823083
MWH-MoK4	Lake Mondsee	47.82975	13.37609	AJ550654	FN823084
MWH-JaK3	Yangtze River	32.11440	118.73201	AJ550657	FN823085
MWH-T1W11	Humble Park Pond 1	31.32588	120.62509	AJ550670	FN823086
MWH-T2W17	Humble Park Pond 2	31.32588	120.62509	AJ550664	FN823087
MWH-HuW1	Tiger Hill Pond	31.33745	120.57617	AJ550666	FN823088
MWH-HuK1	Tiger Hill Pond	31.33745	120.57617	AJ550665	FN823089
MWH-NZ4W10	Lake near Mandeville	-46.00000	168.81667	AM110079	FN823090
MWH-NZ7W17	River near Dunedin	-45.86667	170.50000	AM110083	FN823091
MWH-NZ4W7a	Lake near Mandeville	-46.00000	168.81667	AM110082	FN823092
MWH-NZ4W4	Lake near Mandeville	-46.00000	168.81667	AM110080	FN823093
MWH-Aus1W7	Pond, Sydney	-33.86392	151.21750	AM110081	FN823094
MWH-Aus1W21	Pond, Sydney	-33.86392	151.21750	AM110078	FN823095
MWH-S4W17	Loch Ness	57.24119	-4.52943	AM110087	FN823096
MWH-UK1W16	Unterer Klaffersee	47.29770	13.79374	AM110086	FN823097
QLW-P1DMWA-1	Pond-1	47.73982	13.30167	AJ879783	FN823098
QLW-P2DMWB-1	Pond-2	47.73859	13.30163	FN429654	FN823099
QLW-P2FAT50C-1	Pond-2	47.73859	13.30163	FN429655	FN823100
MWH-P2C2IIa	Pond-2	47.73859	13.30163	FN429656	FN823101
QLW-P1DATA-2	Pond-1	47.73982	13.30167	AJ879801	FN823102
QLW-P2DMWA-1	Pond-2	47.73859	13.30163	FN429657	FN823103
QLW-P1FAT50C-4	Pond-1	47.73982	13.30167	AJ879778	FN823104
MWH-LF2-54b	Lake Loosdrecht	52.20442	5.08127	AJ964893	FN823108
MWH-Gad-W7	Gaddtjärnen	59.86083	15.17944	AM110096	FN823109
MWH-Gad-W5	Gaddtjärnen	59.86083	15.17944	AM110097	FN823110
MWH-Mekk-C3	Mekkojärvi	61.23089	25.14203	AM397065	FN823111
MWH-Mekk-A1	Mekkojärvi	61.23089	25.14203	AM110091	FN823112
MWH-Valk-D1	Valkea-Kotinen	61.23333	25.06667	AM110089	FN823116
MWH-Valk-A4	Valkea-Kotinen	61.23333	25.06667	AM110090	FN823117
MWH-Mekk-B1	Mekkojärvi	61.23089	25.14203	AM110088	FN823113
MWH-Mekk-A6	Mekkojärvi	61.23089	25.14203	AM110094	FN823114
MWH-Svant-W18	Svarttjärnen	59.89000	15.25667	AM110095	FN823118
MWH-Mekk-D6	Mekkojärvi	61.23089	25.14203	AM397066	FN823115
MWH-Braz-FAM2G	Monjolinho, Pond	-21.98548	-47.88088	AM110113	FN823119
MWH-Braz-FAM2J	Monjolinho, Pond	-21.98548	-47.88088	AM110112	FN823120
MWH-Braz-FAM4A	Lobo-Broa, Reservoir	-22.17847	-47.89534	AM110111	FN823121
MWH-Berg-3C6	Bergerie de l'Arate, Pond	42.20229	8.93246	AM110102	FN823122
MWH-Creno-4B5	Lac de Creno	42.20463	8.94588	AM110105	FN823123
MWH-Creno-3A4	Lac de Creno	42.20463	8.94588	AM397064	FN823124
MWH-Creno-4B4	Lac de Creno	42.20463	8.94588	AM110104	FN823125
MWH-PoolBr-B5	Brown Rock Pool	42.17251	8.89848	AM110107	FN823126
MWH-Jannik3A3	Graben	42.08724	8.71062	AM110099	FN823127
MWH-Jannik3D5	Graben	42.08724	8.71062	AM110100	FN823128
MWH-Jannik1A5	Graben	42.08724	8.71062	AM110101	FN823129
MWH-Teich-2B6	Pond close to San Petru	42.08454	8.73069	AM110109	FN823130
czRimov8-C6	Rimov Reservoir	48.84787	14.48943	FN429658	FN823131
czRimovFAM-D3	Rimov Reservoir	48.84787	14.48943	FN429659	FN823132
MWH-Loch2D3-15	Pond Loch2	11.05648	108.39759	FN429660	FN823133
MT-CBaFAMC5	Crystal Bog Lake	46.00556	-89.60417	FN429661	FN823134
MT-CBbDAM6C2	Crystal Bog Lake	46.00556	-89.60417	FN555148	FN823135
MWH-EgelM1-30-B	Egelsee (M1), nähe Mattsee	47.96655	13.12600	FN429662	FN823136
MWH-LakaW10-4	Lake Jezero Laka	49.11133	13.32872	FN429663	FN823139

MWH-PRW10-1	Lake Prášilské	49.07548	13.40023	FN429664	FN823140
MWH-CNW20-3	Lake Černé	49.18017	13.18463	FN429665	FN823141
MWH-Mlynsky-W1(	Mlynsky Pond	48.71075	14.71193	FN429666	FN823142
MWH-Lacke-12-2	Lacke nahe Elmsee	47.68493	13.95980	FN429667	FN823144
MWH-RechtKolB	Rechteckteich	47.07633	12.99489	FN429668	FN823145
AM-25C3	Lake Gosh	40.71940	45.01573	FN429669	FN823148
MWH-UH2A	Kizekibi swamp	0.38333	32.17861	FN429670	FN823149
MWH-UH2B	Kizekibi swamp	0.38333	32.17861	FN429671	FN823150
MWH-UH11A	Kidera wetland	1.24833	33.04500	FN429672	FN823151
MWH-UH14kl	Budongo Forest creek	1.70833	31.48528	FN429673	FN823152
MWH-UH14B	Budongo Forest creek	1.70833	31.48528	FN429674	FN823153
MWH-UH14E	Budongo Forest creek	1.70833	31.48528	FN429675	FN823154
MWH-UH19D	Lukaya pond	-0.12806	31.91306	FN429676	FN823155
MWH-UH21B	Kagona swamp	-0.34361	31.87500	FN429677	FN823156
MWH-UH21F	Kagona swamp	-0.34361	31.87500	FN429678	FN823157
MWH-UH21G	Kagona swamp	-0.34361	31.87500	FN429679	FN823158
MWH-UH23A	Lyantode pond	-0.56750	30.70028	FN429680	FN823159
MWH-UH23C	Lyantode pond	-0.56750	30.70028	FN429681	FN823160
MWH-UH25C	Kabwohe swamp	-0.58139	30.44806	FN429682	FN823161
MWH-UH25E	Kabwohe swamp	-0.58139	30.44806	FN429683	FN823162
MWH-UH35A	Kyegegwa pond	0.52889	30.94861	FN429684	FN823163
MWH-UH36A	Mubende wetland	0.47528	31.62167	FN429685	FN823164
MWH-UH38A	Lake Victoria, Entebbe	0.06389	32.48000	FN429686	FN823165
MWH-UH38Ckl	Lake Victoria, Entebbe	0.06389	32.48000	FN429687	FN823166
MWH-Feld-100	Lake Feldsee	47.87164	8.03378	FN429688	FN823167
MWH-P3-07-1	Pond-3	47.73766	13.30236	FN429689	FN823106
MWH-P4-07-3	Pond-4	47.73670	13.30214	FN429690	FN823107
MWH-Post2−1	pond Post-2	47.65607	13.39818	FN429691	FN823168
MWH-Post4−6−1	pond Post-4	47.65578	13.39838	FN429692	FN823169
MWH-K35W1	Lake Krottensee	47.78338	13.38693	FN429693	FN823170
MWH-CHAdd	Chalupská slať	48.99835	13.65870	FN429694	FN823171
MWH-Cha5	Chalupská slať	48.99835	13.65870	FN429695	FN823172
es-EL-1	Hondo de Elche	38.17634	-0.75872	FN429696	FN823173
es-MAR-1	Marquesado	40.18750	-1.66722	FN429697	FN823174
es-MAR-2	Marquesado	40.18750	-1.66722	FN429698	FN823175
es-MAR-3	Marquesado	40.18750	-1.66722	FN429699	FN823176
es-MAR-4	Marquesado	40.18750	-1.66722	FN429700	FN823177
es-GGE-1	Grande de Gredos	40.25361	-5.27611	FN429701	FN823178
MWH-Tro5−4−8	pond Trög5	47.24989	13.26532	FN429702	FN823179
MWH-Tro8−2−5−gr	pond Trög8	47.24989	13.26532	FN429703	FN823180
MWH-Groes-1−1	Grösster Teich	47.07677	12.99543	FN429704	FN823181
MWH-Unterer−4A−	Unterer Teich	47.07752	12.98887	FN429705	FN823182
MWH-Tro8−2−9	pond Trög8	47.24989	13.26532	FN429710	FN823183
MWH-Tro5−3−9	pond Trög5	47.24989	13.26532	FN429711	FN823185
MWH-Tro6−4−2	pond Trög6	47.24989	13.26532	FN429712	FN823186
MWH-Tro7−1−4	pond Trög7	47.24989	13.26532	FN429713	FN823187
MWH-Recht1	Rechteckteich	47.07616	12.99489	FN429706	FN823146
MWH-Groes-2−3	Grösster Teich	47.07677	12.99543	FN429707	FN823188
MWH-KleinIns−1B-	Kleiner Inselteich	47.07546	12.99138	FN429708	FN823189
MWH-Recht3	Rechteckteich	47.07616	12.99489	FN429709	FN823147
MWH-Weng1-1	Wengmoor	47.92785	13.17628	FN429716	FN823191
MWH-Hall2	Hallstättersee	47.56374	13.66021	FN429717	FN823192
MWH-Hall5	Hallstättersee	47.56374	13.66021	FN429718	FN823193
MWH-Hall10	Hallstättersee	47.56374	13.66021	FN429719	FN823194
MWH-Gerlos1-1	Gerlosplatte, Lacke1	47.23551	12.15430	FN429720	FN823195
MWH-Gerlos4-1	Gerlosplatte, Lacke4	47.23487	12.15421	FN429721	FN823196
MWH-Gerlos5-1	Gerlosplatte, Lacke5	47.23478	12.15392	FN429722	FN823197
MWH-Gerlos6-1	Gerlosplatte, Lacke6	47.23443	12.15428	FN429723	FN823198
MWH-EgelM2-6	Egelsee (M2), near Mattsee	47.96233	13.12470	FN429724	FN823137

MWH-EgelM2-3	Egelsee (M2), near Mattsee	47.96233	13.12470	FN429725	FN823138
LimPoW16	Lake LimnoPolar	-62.64825	-61.10508	HG421771	HG421822
Klost1-W25	Klost1, Klosterheden	56.46777	8.32623	FN429726	FN823199
Klost2-W16	Klost2, Klosterheden	56.47118	8.29387	FN429727	FN823200
Nonnen-W13	Nonnenmattweiher	47.79453	7.79897	FN429728	FN823201
Nonnen-W15	Nonnenmattweiher	47.79453	7.79897	FN429729	FN823202
Ross1-W21	pond Ross1	47.86167	13.47694	FN429730	FN823203
Ross2-W14	pond Ross2	47.86219	13.47596	FN429731	FN823205
Ross6-W10	pond Ross6	47.86744	13.47568	FN429732	FN823206
MWH-Tro8-08W11	pond Trög8	47.24989	13.26532	FN429714	FN823184
MWH-Adler-W1	pond Adlerlacke	47.37256	12.09273	FN429734	FN823207
MWH-Tro7-08W8	pond Trög7	47.24989	13.26532	FN429715	FN823210
MWH-Adler-W8	pond Adlerlacke	47.37256	12.09273	FN429735	FN823208
Ross1-W9	ditch Ross1	47.86167	13.47694	FN429733	FN823204
MWH-Salz1-W7	pond Salz1	47.29254	12.11094	FN429737	FN823211
MWH-Lett3-08W15	pond Letten3	47.36832	12.97123	FN429738	FN823212
MWH-Adler-W21	pond Adlerlacke	47.37256	12.09273	FN429736	FN823209
MWH-Illinger-2A	pond Illinger2	47.72151	13.34776	FN429739	FN823213
SM1-W8	Schwarzes Moos 1	48.87219	14.98022	FN429740	FN823217
Eve-W11	Everglades, Shark Valley	25.74708	-80.76731	FN429741	FN823215
P1-05-14	Pond-1	47.73982	13.30167	FN556008	FN823105
MWH-GrInsel-1	pond Großer Inselteich	47.07580	12.99057	FN556009	FN823190
SM1-W1	pond Schwarzes Moos 1	48.87219	14.98022	FN825821	FN823214
UB-Somero-W24	Lake Somero	-62.65374	-61.12373	HG421772	HG421823
UB-Maderos-W3	Lake Maderos	-62.66300	-61.17699	HG421773	HG421824
UB-Maderos-W12A	Lake Maderos	-62.66300	-61.17699	HG421774	HG421825
UB-Refugio-W16	Lake Refugio	-62.66319	-61.01180	HG421775	HG421826
UB-Chica-W4A	Lake Chica	-62.65750	-61.10917	HG421776	HG421827
UB-Chica-W24	Lake Chica	-62.65750	-61.10917	HG421777	HG421828
UB-Juka-W19	Jukajärvi	61.89200	27.82167	FN825822	FN823216
UB-Kaiv-W7	Kaivoslampi	63.33542	28.83206	FN825823	FN823218
UB-Lampi-W7	Lampi near Venejärvi	63.95392	29.50619	FN825824	FN823219
UB-Raua-W9	Rauanjärvi	63.10422	29.29133	FN825825	FN823220
UB-Siik-W21	Siikajärvi	63.21508	28.39342	FN825826	FN823221
UB-Siik-W7	Siikajärvi	63.21508	28.39342	FN825827	FN823222
UB-Tiil-W10	Tiilikka	63.64906	28.31192	FN825828	FN823223
UB-Vapi-W8	Valkealampi	63.31519	28.87431	FN825829	FN823224
UB-Vilj-W20	Viljus	61.91844	28.03544	FN825830	FN823225
UB-Mula-W16	Mula	62.29283	27.85300	FN825831	FN823226
UB-Rupi-W24	pond near Rupiki (nameless)	63.49322	28.83349	FN825832	FN823227
UB-Rupi-W1	pond near Rupiki (nameless)	63.49322	28.83349	FN825833	FN823228
UB-Blato-W12	Cervene Blato	48.86010	14.81168	FN825836	FN823143
UB-Maderos-W6	Lake Maderos	-62.66300	-61.17699	HG421791	HG421842
UB-LimW2	Lake LimnoPolar	-62.64825	-61.10508	HG421792	n.d.
UB-Domo-W1	Lake Domo	-62.64806	-60.98207	HG421793	HG421844
UB-Kamb-W5	Ankor Wat, ditch	13.41245	103.86023	FN825837	FN823231
UB-Kamb-W7b	Ankor Wat, ditch	13.41245	103.86023	FN825838	FN823232
UB-Kamb-W7a	Ankor Wat, ditch	13.41245	103.86023	FN825839	FN823233
UB-Piko-W3	Pien-Koikka	63.13242	27.80925	FN825834	FN823229
UB-Petae-W23	Petäisjoki	63.32886	28.82089	FN825835	FN823230
UK-BL3-W12	Blindensee	48.07439	9.53901	FR682912	FR682909
UK-BL2-W11	Blindensee	48.07439	9.53901	FR682911	FR682910
UK-Kesae-W10	Kesälampi	63.24950	26.97603	HG421778	HG421829
UK-Long2-W17	Longyearbyen Pond 2	78.21857	15.69481	HG421821	n.d.
UK-Maja-W20	Majalampi	63.21125	26.82685	HG421779	HG421830
UK-Pondora-W13	Pandora pond	63.47487	27.42527	HG421780	HG421831
UK-Pondora-W15	Pandora pond	63.47487	27.42527	HG421781	HG421832
UK-Piela-W12	Pielavesi	63.35410	26.42725	HG421782	HG421833
UK-Piela-W13	Pielavesi	63.35410	26.42725	HG421783	HG421834

UK-Ruja-W24	Rujalampi	63.23958	26.98507	HG421784	HG421835
UK-Tiil2-W2	Tiilikka	63.64912	28.31185	HG421785	HG421836
UK-Gri1-W3	pond Grimselpass, Gri1	46.55339	8.33544	HG421786	HG421837
UK-Kesae-W13	Kesälampi	63.24950	26.97603	HG421787	HG421838
UK-Loeyt-W23	Löytäna	63.40243	26.85395	HG421788	HG421839
MG-4-Mela-C2	not known	63.09506	30.12842	HG421789	HG421840
MG-4-Mela-E1	not known	63.09506	30.12842	HG421790	HG421841
MG-4-Mela-F3	not known	63.09506	30.12842	n.d.	HG421843
MG-8-Puso-13	not known	62.95628	29.87550	HG421794	HG421845
MG-27-Goln-C1	not known	70.06783	29.98053	HG421795	HG421846
MG-27-Goln-C3	not known	70.06783	29.98053	HG421796	HG421847
MG-52-Enon2-B3	not known	68.34889	24.13167	HG421797	HG421848
MG-52-Enon2-B4	not known	68.34889	24.13167	HG421798	HG421849
MG-63-Kera-F1	Keräntöjärvi	67.66008	22.90206	HG421799	HG421850
MG-65-Swed-E4	not known	66.77272	23.14856	HG421800	HG421851
MG-6-Vaara-E2	Väärälampi	63.09731	29.66608	HG421801	HG421852
MG-7-Harju-B2	not known	63.00797	29.75319	HG421802	HG421853
MG-8-Puso-B2	not known	62.95628	29.87550	HG421803	HG421854
MG-22-Aci1-F1	not known	69.13086	27.76061	HG421804	HG421855
MG-25-Pas1-D2	not known	69.44558	29.90306	HG421805	HG421856
MG-25-Pas1-F1	not known	69.44558	29.90306	HG421806	HG421857
MG-28-Ekke-A2	not known	70.07119	30.11672	HG421807	HG421858
MG-43-Slet-D2	not known	71.09475	28.18311	HG421808	HG421859
MG-43-Slet-F1	not known	71.09475	28.18311	HG421809	HG421860
MG-58-Narv-E3	not known	68.53425	17.29506	HG421810	HG421861
MG-Unter1-9	Lake Egelsee (near Attersee)	47.83273	13.50404	HG421811	HG421862
MG-Unter2-12	Lake Egelsee (near Attersee)	47.83273	13.50404	HG421812	HG421863
MG-Unter2-18	Lake Egelsee (near Attersee)	47.83273	13.50404	HG421813	HG421864
MG-5-Ahmo-C2	not known	63.08494	29.58561	HG421814	HG421865
MG-58-Narv-B1	not known	68.53425	17.29506	HG421815	HG421866
MG-42-Nopo-D3	not known	71.08494	28.16397	HG421816	HG421867
MG-65-Swed-B2	not known	66.77272	23.14856	HG421817	HG421868
MG-54-Pals-D4	not known	68.72422	21.42044	HG421818	HG421869
