# Supplementary material for: Global phylogeography of pelagic Polynucleobacter bacteria: Restricted geographic distribution of subgroups, isolation by distance and influence of climate
Source: Environ Microbiol. 2014 Jul 15;17(3):829–40. doi: 10.1111/1462-2920.12532 (PMC4361717; doi:10.1111/1462-2920.12532)
Supplement: Table S2 — Detection of Polynucleobacter necessarius ssp. asymbioticus subgroups by reverse line blot hybridization (RLBH) in habitats located in Uganda. [file emi0017-0829-sd4.pdf]

Supplementary Materials Table S2. Detection of *Polynucleobacter necessarius* ssp. *asymbioticus* subgroups by Reverse Line Blot Hybridization (RLBH) in habitats located in Uganda. Only results for those 22 samples, which yielded PCR products are shown. For details on the methods and RLBH probes see Jezbera et al., 2011.

Site	F1	F2	F4	F5	F10	F11	F12	F13n	F14	F15	F15-1	F16	F17
Kabwohe wetland, Mbarara								vs	vw				
Kizekibi swamp								vs					
Kagona wetland, Masaka								vs	vw			vw	
Kigwe, stream wetland								vs	vw				
Kasana wetland								vs	vw				
Budongo forest swamp								vs	vw				
Lake Nabugabo						vs	vs	vs					
Kagoma, Jinja swamp													
Buwera swamp, Kamuli						vw		vs		vw			
Kiyanja swamp								vs	vw	vw			
Lwera wetland, Lukaya								vs	vw			vw	
Kyazanga, Masaka								vs					
Kinyara small river							vs	vs					
Musuno swamp, Kamuli						vw		vs		vw		vw	
Masindi port wetland						vw		vs	vw			vw	
Nyakibere crater Lake													
Batesi, Lake Wamala						vw	vw	vs					
Nkuruba crater Lake													
Butiaba, Lake Albert								vs		vw			
Kasenyi, Lake George								vs					
Kazinga channel, Bushenyi								vs	vw				
Katwe, Lake Edward								vw					

Hybridization intensity

very strong	vs
strong	
average	
weak	
very weak	vw
no signal	
