# Supplementary material for: Global phylogeography of pelagic Polynucleobacter bacteria: Restricted geographic distribution of subgroups, isolation by distance and influence of climate
Source: Environ Microbiol. 2014 Jul 15;17(3):829–40. doi: 10.1111/1462-2920.12532 (PMC4361717; doi:10.1111/1462-2920.12532)
Supplement: Table S3 — Antarctic lakes investigated for the presence of the Antarctic clade. [file emi0017-0829-sd5.pdf]

Supplementary Materials Table S3. Antarctic lakes investigated for the presence of the Antarctic clade (see Fig. 1b) by isolation of strains and/or nested PCR with specific primers. All lakes are located in Byers Peninsula (Livingston Island) or in Hope Bay (Antarctic Peninsula), all in the maritime Antarctica. Note that not all samples were investigated by both methods. The lakes were sampled in 2004, 2007, and/or 2009 during austral summer (January and February)

Lake	Site	Habitat type	Temp. (°C)	pH	Conductivity [µS cm^{-1}]	Absorption (250 nm)	Geographic Coordinates Latitude	Longitude	Detection of Antarctic Clade	Method	Year
Lake Limnopolar	B.P.	Antarctic mid-depth lake (inland)	4.5	7.5	67	0.0103	62°38'53"S	61°06'18"W	pos.	Cult., PCR	2004, 07, 09
Lake Somero	B.P.	Antarctic shallow lake (inland)	5.4	7.2	70	0.0278	62°38'54"S	61°06'41"W	pos.	Cult., PCR	2007, 09
Lake Maderos	B.P.	Antarctic shallow lake (coastal)	3.0	7.4	257	0.0402	62°39'46"S	61°10'37"W	pos.	Cult.	2009
Lake Refugio	B.P.	Antarctic shallow lake (coastal)	4.4	8.5	130	0.0649	62°39'47"S	61°00'42"W	pos.	Cult., PCR	2007, 09
Lake Chica	B.P.	Antarctic mid-depth lake (inland)	5.7	6.8	45	0.0206	62°39'27"S	61°06'33"W	pos.	Cult., PCR	2004, 27, 09
Lake Domo	B.P.	Antarctic mid-depth lake (inland)	2.8	7.0	26	0.0120	62°38'53"S	60°58'55"W	pos.	Cult.	2009
Lake Midge	B.P.	Antarctic deep (9 m) lake (inland)	4.6	6.9	68	0.0128	62°37'49"S	61°05'42"W	pos.	PCR	2007
Lake Chester	B.P.	Antarctic mid-depth lake (inland)	5.6	6.9	52	0.0258	62°38'09"S	61°05'55"W	pos.	PCR	2007
Lake Turbio	B.P.	Antarctic mid-depth lake (inland)	5.1	6.6	58	0.0287	62°39'05"S	61°05'16"W	pos.	PCR	2007
Lake Escondido	B.P.	Antarctic mid-depth lake (inland)	4.1	6.7	52	n.a.	62°38'36"S	61°03'34"W	pos.	PCR	2007
Lake Kokotxas	B.P.	Antarctic shallow lake (inland)	4.0	7.0	51	n.a.	62°35'52"S	61°10'02"W	pos.	PCR	2007
Lake Boeckella	H.B.	Antarctic mid-depth lake (inland)	0.2	6.5	19	n.a.	63°23'59"S	57°00'00"W	pos.	PCR	2004
Lake Encantado	H.B.	Antarctic shallow lake (coastal)	0.1	6.6	67	n.a.	63°24'36"S	57°02'24"W	pos.	PCR	2004
Lake Esperanza	H.B.	Antarctic mid-depth lake (coastal)	1.0	6.4	91	n.a.	63°24'36"S	57°01'48"W	neg.	PCR	2004
Lake Flora	H.B.	Antarctic shallow lake (coastal)	3.0	6.3	32	n.a.	63°24'36"S	57°02'24"W	neg.	PCR	2004
Pingüi Pond	H.B.	Antarctic pond (coastal)	0.8	7.1	1860	n.a.	63°23'59"S	57°00'36"W	neg.	PCR	2004

B.P., Byers Peninsula, Livingston Island; H.P., Hope Bay, Antarctic peninsula; Temp., water temperature; pos., positive detection; neg., negative detection; n.a. = not available
